# Adverse reactions from consumption of oral rabies vaccine baits in dogs in Finland

**DOI:** 10.1186/s13028-016-0234-3

**Published:** 2016-09-15

**Authors:** Tiina Nokireki, Martti Nevalainen, Liisa Sihvonen, Tuija Gadd

**Affiliations:** 1 Finnish Food Safety Authority Evira, Mustialankatu 3, 00790 Helsinki, Finland; 2 Finnish Medicines Agency Fimea, P.O. Box 55, 00034 Fimea, Helsinki Finland; 3 Department of Veterinary Biosciences, Faculty of Veterinary Medicine, University of Helsinki, P.O. Box 66, 00014 Helsinki, Finland

**Keywords:** Adverse reaction, Oral rabies vaccine, Hunting dog, SAG 2

## Abstract

**Background:**

Oral rabies vaccination of wildlife has effectively reduced the incidence of rabies in wildlife and has led to the elimination of rabies in large areas of Europe. The safety of oral rabies vaccines has been assessed in both target (red fox and raccoon dog) and several non-target species.

**Case presentation:**

Since 2011, the competent authority in Finland has received a few reports of dogs experiencing adverse reactions that have been assumed to be caused by the consumption of baits containing oral rabies vaccine. The dogs usually exhibited gastrointestinal symptoms (vomiting, inappetence, constipation or diarrhoea) or behavioral symptoms (restlessness, listlessness and unwillingness to continue hunting).

**Conclusions:**

Nevertheless, these adverse reactions are transient and non-life threatening. Even though the adverse reactions are unpleasant to individual dogs and their owners, the benefits of oral rabies vaccination clearly outweigh the risks.

## Background

Rabies virus (RABV) infection is maintained in several species of wildlife, but rabid dogs are the most important source of human rabies infections, especially in Asia and Africa. Historically, the dog has been the most important host for RABV in Europe, but during the first half of the 20th century dog rabies was eliminated in most parts of Europe. RABV emerged in red foxes (*Vulpes vulpes*) in the Kaliningrad area at the beginning of the 1940s and spread within a few decades across Central and Western Europe [1]. In 1978, the first oral rabies vaccination campaign for wildlife was conducted in Switzerland, followed by other European countries. Oral rabies vaccination has effectively reduced the incidence of rabies in wildlife during the past three decades in 24 countries and has led to its elimination over large areas of Europe [2].

Finland had been free of rabies since 1959, but the disease reappeared in 1988–1989 in an area of 1700 km^2^ in southeast Finland. Altogether, 66 cases were recorded, mostly in raccoon dogs (*Nyctereutes procyonoides*), but also in red foxes and domestic animals. A field trial of oral vaccination was initiated in 1988 using SAD B19 bait, and the outbreak was contained after three oral vaccination campaigns and compulsory vaccination of dogs in the outbreak area [4]. Finland was declared rabies-free again in 1991. However, because of concerns of rabies re-emergence over the eastern and southeastern borders, oral rabies vaccination campaigns have been carried out in Finland every year since 1991. Rabies is endemic in Russia with 3371 cases reported in 2015 in domestic and wild animals. The Baltic countries have only in recent years been able to reduce the number of cases and eradicate rabies from their territory [3]. In Finland, the vaccination area, the vaccine used, and the number of vaccination campaigns per year has varied over time. Since 2010, the SAG 2 vaccine (Rabigen, Virbac, France) has been used. In 2011, after the Russian federation authorities informed about rabies cases in Karelia, Finland extended the vaccination zone from about 5000 km^2^ to 10,000 km^2^ by both widening the zone and extending it more north. The number of vaccine baits distributed has varied from 160,000 to 360,000 vaccine baits per year.

**Fig. 1:**
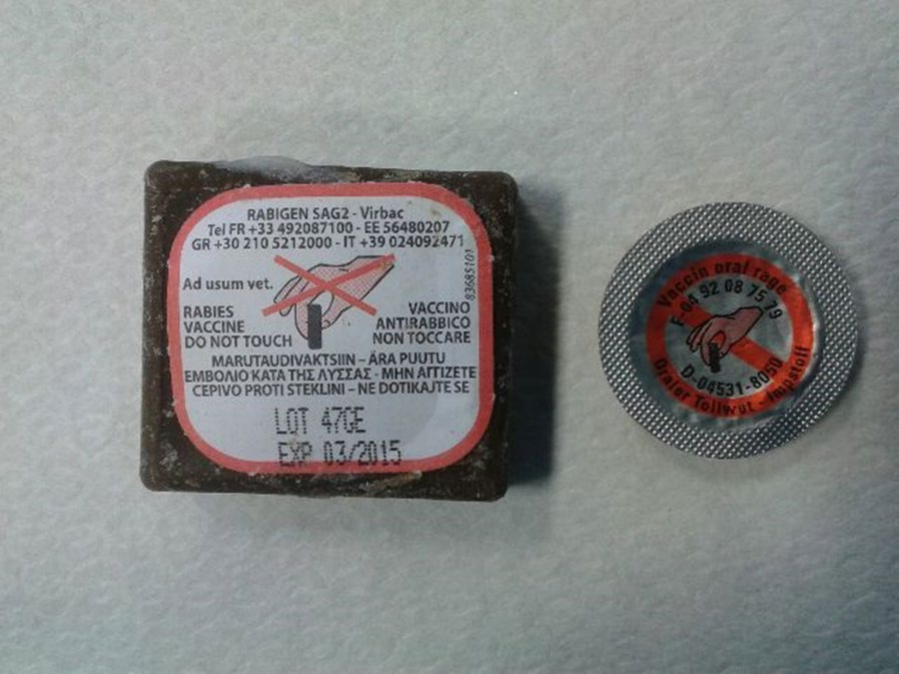
The oral rabies vaccine bait and the PVC/aluminium sachet

SAG 2 is a modified-live rabies vaccine. The strain was selected from SAD Bern in a two-step process of amino acid mutation. The liquid suspension containing the vaccine is presented within a PVC/aluminium sachet. The sachet is coated with bait matrix made of fat and fish ingredients [5] (Fig. 1). The safety of the vaccine has been evaluated in both target (red fox and raccoon dog) and several non-target species [6–8]. The administration of the vaccine at 10 times the recommended dosage induced no adverse effects in raccoon dogs [6]. Since this vaccine presentation contains tetracycline as a biomarker and traces of gentamycin, occasional hypersensitivity reactions may be observed in domestic animals that have ingested the bait containing oral rabies vaccine [9].

In Finland, oral rabies vaccination takes place in September–October. During autumn, the raccoon dog and fox populations are at their largest and the weather conditions are suitable for the bait distribution [7, 8]. It coincides with the active hunting season, when hunting dogs are also used. While they are roaming freely in the vaccination area, they have the opportunity to consume bait. Before 2011, no adverse reactions were reported to the competent authority. Since 2011, however, the competent authority received a few reports of dogs experiencing adverse reactions that are suspected to have been caused by the ingestion of baits containing oral rabies vaccine.

## Case presentation

**Fig. 2:**
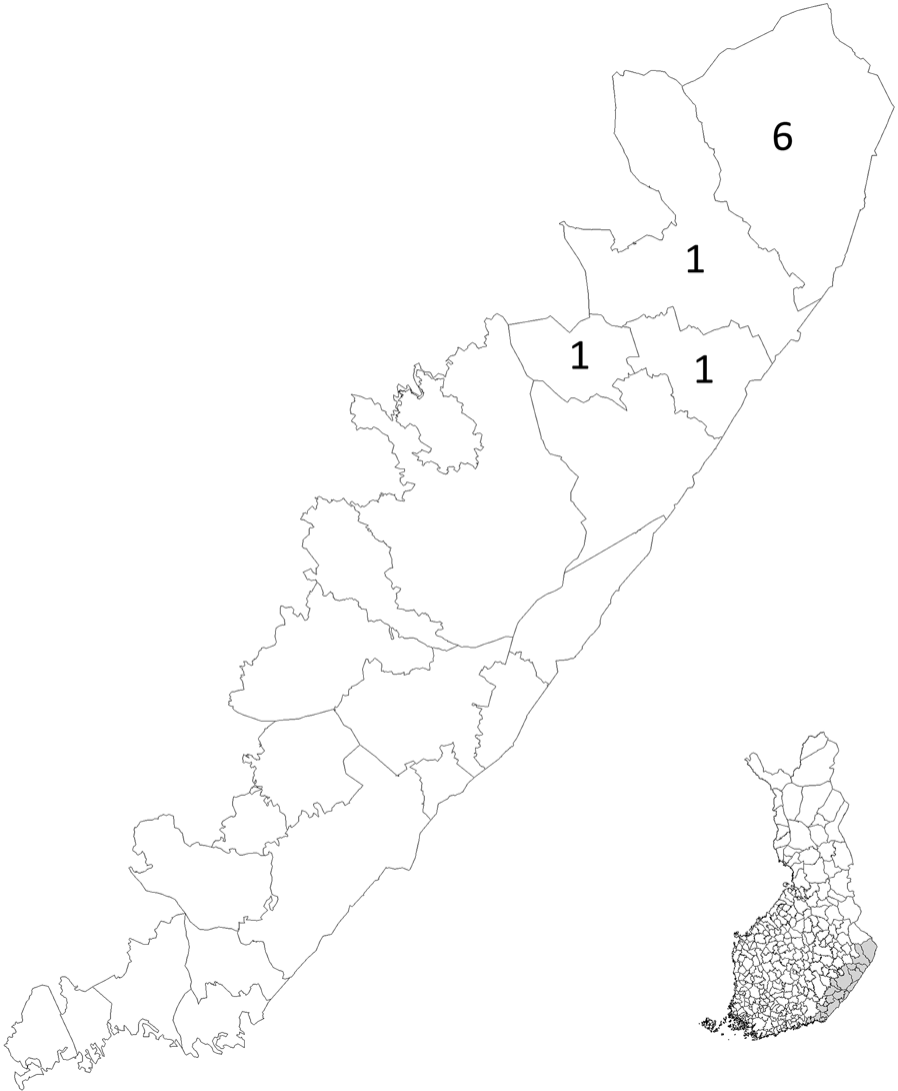
Map presenting the geographical distribution of reported cases in the vaccination area in Finland

During 2011–2014, the competent authorities received nine reports from dog owners or veterinarians about dogs exhibiting symptoms that were associated with the consumption of bait containing oral rabies vaccine during hunting. Reported cases occurred in the northern part of the vaccination area on the southeastern border (Fig. 2). Owners had either seen their dog eat a bait or there had been vaccine sachets in the dog’s vomit. However, the consumption of bait containing the vaccine was not confirmed in all cases. Two cases were considered serious. At least three of these dogs were taken to a veterinarian, and at least one underwent surgery and one endoscopy. The dogs usually exhibited gastrointestinal symptoms (vomiting, inappetence, constipation or diarrhoea) or behavioral symptoms (restlessness, listlessness and unwillingness to continue hunting) (Table 1). In addition to the reported adverse reaction in dogs, a report was received of a child who had been handling and tasting bait, although the child did not experience any adverse effects. Due to these adverse event reports, Section  4.6 of the SPC for the vaccine has been amended by the manufacturer as follows: ‘‘Vomiting due to gastric intolerance (potentially due to the aluminium/PVC sachet as part of the bait vaccine) has been reported in dogs that have accidentally ingested the bait.”

**Table 1:** Adverse events in dogs in 2011-2015 reported to the Finnish competent authority due to suspected consumption of bait containing oral rabies vaccine

Case id	Year	ABON^a^	Dog	Municipality	Clinical description by the owner	Examination and treatment by veterinarian
Case 1	2011	A	German shorthaired pointer	Ilomantsi	Vomiting (5 folio capsules)	No treatment
Case 2	2011	B	Beagle, 1 year	Rääkkylä	Restlessness, vomiting, listless (vomited a folio capsule)	Symptomatic treatment
Case 3	2012	B	Norwegian elkhound, male	Kitee	Vomiting, inappetence, listless	No treatment
Case 4	2012	B	Finnish spitz, female	Ilomantsi	Listless, emesis	No treatment
Case 5	2012	A	Finnish spitz	Ilomantsi	Listless, emesis, weight loss, anorexia, constipation, haemorrhagic diarrhoea	X-ray, surgery for removal of foreign body
Case 6	2012	A	Jämthund (Swedish Jämthund)	Ilomantsi	Listless, emesis, weight loss, anorexia, constipation, haemorrhagic diarrhoea	No treatment
Case 7	2012	B	Finnish spitz, male	Ilomantsi	Emesis, listless	No treatment
Case 8	2012	O	Finnish spitz, male	Tuupovaara	Hyperactive, confusion, did not hunt for 2 days	No treatment
Case 9	2014	A	German shorthaired pointer, female	Ilomantsi	Vomiting	X-ray, endoscopy

^a^ The causality assessment was carried out using the ABON system described in Volume 9B, Guidelines on Pharmacovigilance for Medicinal Products for Veterinary Use where categories are as follows: *A* probable; *B* possible; *O* unclassifiable/unassessable; *N* unlikely to be product related

## Conclusions

A small number of cases concerning adverse events in dogs due to the consumption of bait containing oral rabies vaccines have been recorded, even though it is likely that not all cases have been reported to the competent authority. Nevertheless, it appears that taking into consideration the quantity of baits containing oral rabies vaccine that has been distributed in the wild, the number of reported adverse events is low. The gastrointestinal symptoms are probably attributable to gastric intolerance following the ingestion of foreign material, i.e., aluminium/PVC sachets. Likewise, the other signs reported such as apathy, lethargy, or on the contrary, restlessness are likely to be related to discomfort caused by the ingestion of multiple baits. According to Cliquet et al. [10], dogs consumed vaccine bait immediately after presentation, while foxes and raccoon dogs were less eager to ingest the bait. Thus, it appears that dogs are by nature more likely to consume several baits than the target species. It is also noteworthy that all other oral rabies baits have the same components. The possibility that the antibiotics present in the bait could cause reactions in some individuals cannot be ruled out. The aminoglycoside antibiotic gentamycin is known to induce acute kidney injury in dogs [11].

It is very important that people who live or hunt in the vaccination area are aware of the bait distribution, since its timing coincides with hunting season. In 2011, the vaccination campaign in Finland was extended to areas that had not been vaccinated before, and all of the adverse event reports came from these areas. It is possible that people living and hunting there reported the adverse reactions more readily than people living in the area where vaccination has been carried out for years. All reports involved hunting dogs as may be expected, because hunting dogs roam freely over large areas of the vaccinated zone. They could easily ingest several baits unnoticed by their owner. Hunting is strenuous exercise for dogs. Slight adverse symptoms may readily affect their overall performance, which is obvious to their owners and reported.

If an owner knows or suspects that a dog has ingested bait, the owner could try to induce vomiting. The dog’s health should be monitored, and in case of apparent serious clinical signs, the owner should contact a veterinarian. If such signs could be related to the vaccine bait consumption, case should be reported to Finnish Medicines Agency Fimea.

We conclude that even though these adverse reactions are unpleasant to individual dogs and their owners, the benefits of oral rabies vaccination clearly outweigh the risks.
